# Predicting variable-length paths in networked systems using multi-order generative models

**DOI:** 10.1007/s41109-023-00596-x

**Published:** 2023-09-22

**Authors:** Christoph Gote, Giona Casiraghi, Frank Schweitzer, Ingo Scholtes

**Affiliations:** 1 Chair of Systems Design, ETH Zurich, Zurich, Switzerland; 2 Chair for Machine Learning for Complex Networks, Julius-Maximilians-Universität Würzburg, Würzburg, Germany; 3 Data Analytics Group, University of Zurich, Zurich, Switzerland

**Keywords:** Graph mining, Sequential pattern mining, Sequence prediction, Supervised learning

## Abstract

Apart from nodes and links, for many networked systems, we have access to data on paths, i.e., collections of temporally ordered variable-length node sequences that are constrained by the system’s topology. Understanding the patterns in such data is key to advancing our understanding of the structure and dynamics of complex systems. Moreover, the ability to accurately model and predict paths is important for engineered systems, e.g., to optimise supply chains or provide smart mobility services. Here, we introduce MOGen, a generative modelling framework that enables both next-element and out-of-sample prediction in paths with high accuracy and consistency. It features a model selection approach that automatically determines the optimal model directly from data, effectively making MOGen parameter-free. Using empirical data, we show that our method outperforms state-of-the-art sequence modelling techniques. We further introduce a mathematical formalism that links higher-order models of paths to transition matrices of random walks in multi-layer networks.

## Introduction

Network models have provided us with important insights into the structure and dynamics of complex systems that consist of many interacting elements. In these models, we represent direct interactions between elements by means of dyadic or, increasingly, polyadic relationships, e.g., directed or undirected links connecting pairs of nodes, hyperedges connecting groups of nodes, or abstract simplicial complexes simultaneously capturing multiple (sub)sets of interacting nodes (Benson et al. 2021; Torres et al. 2021). A key contribution of this *topological* perspective is that it tells us which of a system’s elements can possibly influence each other either *directly* via a relationship, or *indirectly* via sequences of relationships that constitute *paths*.^1^ While the topology of a network tells us which paths are possible in principle, we nowadays often directly observe paths, i.e., we have access to data capturing time-ordered sequences of nodes that interact with each other or that are traversed by a process. Examples include time-stamped interactions capturing how information (Dai et al. 2014; West and Leskovec 2012; Arlitt and Jin 2000; Cadez et al. 2000) or infectious diseases (Chai and Pavlou 2016; Wang and Wu 2018; Jo et al. 2021) propagate along sequences of social actors, data about sequences of proteins, genes, or cells that lead to a specific biological function (Olson 2006; Shapira et al. 2009; Karlebach and Shamir 2008), or data on the flow of goods in logistics networks or supply chains (Kim et al. 2015; Li and Zobel 2020; Pavlov et al. 2019). Methods that help us to model and understand sequential patterns in such data, especially those that cannot be explained by the topology of the underlying network, bear the promise to fundamentally advance our understanding of complex systems (Schwarze and Porter 2021). As such, path-centric models constitute an interesting class of *higher-order networks* that model both the temporal and topological dimension of complex systems (Lambiotte et al. 2019). Moreover, the ability to accurately predict paths in networks such as travel itineraries in transportation systems  (Hackl et al. 2018; RITA 2014; Transport for London 2014) or user navigation on eCommerce websites (Montgomery et al. 2004; Bollen et al. 2009; Hui et al. 2009; Brodley and Kohavi 2000) can help us to more effectively manage travel disruptions, break infection paths, recommend related products, or predict online purchases.

Despite this importance, the modelling of patterns in data on paths in networks is still an open challenge. To address it, we must *simultaneously* account for three characteristics of real-world data: (i) we typically have large collections of (short) paths with variable lengths, (ii) each of those paths consists of a temporally ordered node sequence, and (iii) possible node sequences are constrained by an underlying network topology, i.e. two subsequent nodes *u*, *v* can only occur if a link (*u*, *v*) exists. Previously used modelling frameworks have, at most, accounted for two of these three characteristics at the same time. Variable length sequences are typically studied by means of sequence modelling algorithms (Tax et al. 2020; Fournier-Viger et al. 2017). Examples are Petri nets (Weijters and Ribeiro 2011; Augusto et al. 2017), Markov models (Deshpande and Karypis 2004; Gündüz and Özsu 2003; Bernhard et al. 2016; Pitkow and Pirolli 1999; Cleary and Witten 1984; Chierichetti et al. 2012; Singer et al. 2014; Benson et al. 2017), decision trees (Gueniche et al. 2015; Buijs et al. 2012; Leemans et al. 2018), spectral learning (Balle et al. 2014), as well as neural networks and automata (Tax et al. 2020). Such algorithms do not account for the underlying network constraining these sequences. Instead, network models are used to capture topological patterns (Padmanabhan and Mogul 1996; Laird and Saul 1994; Peixoto and Rosvall 2017; West and Leskovec 2012; Brockmann and Helbing 2013) but neglect patterns in the *sequence* of traversed nodes (Xu et al. 2016; Scholtes 2017). Recent works on higher-order network models have taken a first step towards combining the advantages of sequence modelling algorithms and standard network models. They have improved our ability to cluster and rank nodes (Rosvall et al. 2014; Xu et al. 2016; Scholtes 2017), detect anomalies (LaRock et al. 2019) and open new perspectives for network embedding (Saebi et al. 2019; Belth et al. 2019). However, they still do not account for the finite and variable lengths of paths, which limits our ability to accurately predict variable-length paths in networks.

**Fig. 1:**
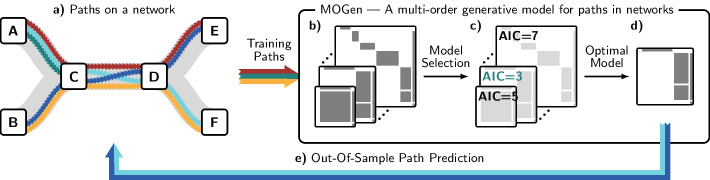
**a** Five colour-coded paths between a set of six nodes (A–F) in a networked system. We split the paths into a training set (  ,  ,  ) and a validation set (  ,  ). The topology of the networked system—depicted in grey—limits which transitions can exist. All paths start in A or B. They are of variable length as they end either after a single transition in C, or after three transitions in E or F. Consider aiming to predict if a path arriving in C continues to D or ends. Only paths starting in A can end in C, whereas all paths starting in B continue to D. Hence, to make an informed prediction, we need to account for the temporal ordering of transitions, i.e., we require memory recording the sequence of visited nodes before arriving at C. **b** To fit MOGen, we estimate a set of models encoding the observed transitions with different maximum memory lengths. **c** We apply MOGen’s model selection algorithm to determine the optimal maximum order. **d** We find the optimal model for the given data. **e** We use this optimal model to predict the paths in the validation data

Addressing this gap, our work makes the following contributions: First, we introduce MOGen, a method to learn compact models of large collections of paths that account for both the temporal ordering and variable-length of paths, as well as underlying network constraints. Second, we show how the models generated by MOGen can be represented in terms of block adjacency and transition matrices, using a previously unknown relation between higher-order network models and matrix representations of multi-layer networks. Third, we propose an efficient model selection algorithm that yields an optimal model to encode sequential patterns in a given set of paths. We evaluate this algorithm in empirical data and show that it yields generalisable models that neither under- nor overfit sequential patterns. Finally, we show that MOGen accurately predicts paths in networks. Unlike existing algorithms, our method supports both predicting the next node based on a prefix of varying length (next-element prediction) and the out-of-sample prediction of full paths based on a training set. We apply our method to six data sets on human clickstreams and passenger itineraries in transportation networks and demonstrate its superior performance compared to state-of-the-art sequence modelling algorithms and higher-order network models. An overview of our method is shown in Fig. 1.

## Results

We now present the four key results of the article.

### Data sets

To obtain these results, we evaluate MOGen in six empirical data sets containing (i) user clickstreams on the Web, and (ii) travel itineraries of passengers in a train and an airline network. Here, BMS1 records clickstreams of customers of the web retailer *Gazelle.com* (Brodley and Kohavi 2000). Similarly, FIFA captures clickstreams of users of the ’98 FIFA World Cup website (Arlitt and Jin 2000), and MSNBC contains clickstreams of page categories on the MSNBC news website (Cadez et al. 2000). Our final clickstream data set WIKI records navigation paths of users in the Wikipedia article graph, who were playing the game Wikispeedia (West and Leskovec 2012). For our second category, AIR contains flight itineraries of passengers travelling on routes between US airports in 2001 (RITA 2014), and TUBE captures itineraries of London Tube passengers (Transport for London 2014). The raw data for all data sets are freely available online.^2^ We provide extended summary statistics in Table 1.

**Table 1:** Summary statistics for the six data sets used to validate MOGen.

	Paths		Nodes on path				Network topology		
	Total	Unique	Mean	Median	Min	Max	Nodes	Links	Density (%)
BMS1	59,601	18,473	2.51	1	1	267	497	15,387	6.24
FIFA	20,450	20,053	36.24	31	9	100	2990	73,530	0.82
MSNBC	31,790	30,247	13.33	11	9	100	17	270	93.43
WIKI	76,193	68,784	6.25	5	1	435	4179	69,800	0.40
AIR	286,810	60,228	4.19	5	2	14	175	1598	5.24
TUBE	4,295,731	32,313	7.86	7	2	36	276	663	0.87

BMS1: online retailer clickstreams (Brodley and Kohavi 2000); FIFA: FIFA World Cup 98 server logs (Arlitt and Jin 2000); MSNBC: news website clickstreams (Cadez et al. 2000); WIKI: Wikispeedia clickstreams (West and Leskovec 2012); AIR: US flight itineraries (RITA 2014); TUBE: London Tube itineraries (Transport for London 2014)

### Next-element prediction performance

First, we assess MOGen’s performance for the task of next-element prediction. *Next-element prediction* seeks to predict the next element(s) in a given ordered sequence based on observed data. This problem, typically addressed with sequence modelling techniques, occurs in a number of applications, e.g., recommender systems (Frias-Martinez et al. 2002), speculative Web caching (Bestavros 1995), or purchase prediction in eCommerce (Kraus and Feuerriegel 2019). Uniquely, MOGen considers both the underlying network and the sequential patterns in path data. As a result, MOGen outperforms state-of-the-art sequence modelling algorithms in next-element prediction tasks.

Given a prefix of *n* nodes $(v_{t-n-1},..., v_{t-1})$ traversed by a path, we predict the next step of the path. This next step can either be the next visited node $v_{t}$, or the end of the path $\dagger$. We include the prediction of path ends in our evaluation as the prediction of terminal nodes is crucial in variable-length path data, e.g., to predict where users exit a website, where passengers end their itinerary, or whether a purchase is made in an online shop.

We compare the performance of MOGen to state-of-the-art sequence modelling algorithms. Specifically, we compare against the higher-order Markov chain based method AKOM (Pitkow and Pirolli 1999), which has shown the highest performance in the review paper (Tax et al. 2020). In addition, as the authors of (Gueniche et al. 2015) show that CPT+ outperforms AKOM in terms of prediction accuracy, we also include CPT+ in our set of baselines methods. Further, we consider predictions based on a higher-order network with a single layer (NET), the multi-order graphical model (MOM) introduced in Scholtes (2017), and a naive random baseline (RND) that predicts the next node based on the relative frequency of nodes in the training data.

To compare the models, we create a 90%/10% train-validation split for each set of path data. We fit the models based on the training paths. From the validation paths, we generate all possible prefix-target combinations up to prefix length $N=6$. Specifically, given a target $v_t$ on path $(v_1,..., v_{l}, \dagger )$ in the validation data, we enumerate all possible prefixes $\left\{ (v_{t-n},..., v_{t-1})\right\} _{n\in [1,6]}$, where *n* is the length of the prefix. Repeating this procedure for all targets on all paths in the validation data yields a set of prefix-target combinations against which we evaluate next-element prediction performance in terms of the *cross-entropy loss function*. We provide further details on the experimental setup for the next-element prediction, including additional information regarding the baseline models, the parameter selection, and the cross-entropy loss function in the Materials and Methods section.

**Table 2:** Next-element prediction performance of all models

	BMS1	FIFA	MSNBC	WIKI	AIR	TUBE
(a) Cross-entropy loss [bit] for prefixes up to length 6						
MOGen	**6.14**±**.07**	**7.32**±**.12**	**2.85**±.**02**	**7.90**±.**06**	**4.20**±.**01**	**1.81**±.**00**
AKOM	19.60±.30	8.53±.11	6.01±.04	14.40±.05	13.27±.01	7.09±.00
CPT+	19.33±.28	10.66±.02	7.33±.04	17.42±.02	14.84±.01	10.42±.00
NET	19.60±.30	8.69±.11	6.13±.04	14.40±.05	13.34±.01	7.57±.00
MOM	19.60±.30	8.51±.12	6.01±.04	14.40±.05	13.20±.01	6.34±.01
RND	19.68±.24	9.63±.04	6.73±.03	15.79±.03	14.11±.01	11.94±.00
(b) MOGen: detected and best performing maximum order						
detected	1	1	2	1	2	6
best	1	1	2	1	2	6

(a) Mean and standard deviation of the cross-entropy loss over five train-validation splits. For each data set, the result of the best performing model is highlighted in bold. (b) Maximum order of the best performing MOGen model and the order detected by model selection

In Table 2a, we report the mean cross-entropy loss (in bits) for the six empirical data sets and the six prediction methods described above averaged over five train-validation splits. For each algorithm, we report the performance for the parameters that yield the smallest cross-entropy loss. The results show that MOGen outperforms all other methods. In most of the data sets, we further observe a considerable difference in the cross-entropy loss function. We conjecture that this large difference in performance is due to the fact that, different from other methods, MOGen (i) explicitly models varying-length paths in a network, and (ii) is able to predict the termination of paths. This highlights the main contribution of our work, which is a modelling framework that accounts specifically for the characteristics of data on paths in networks. We observe the smallest cross-entropy loss for MSNBC, AIR, TUBE, which we obtain thanks to the—relative to the size of the underlying network—large number of observed paths. FIFA, WIKI, and BMS1 generally yield the largest cross-entropy loss across all methods, which come from the large network topologies and a relatively small number of observations, which hinders a reliable detection of generalisable sequential patterns. Table 2 reports the prediction performance of methods across multiple prefix lengths.

**Fig. 2:**
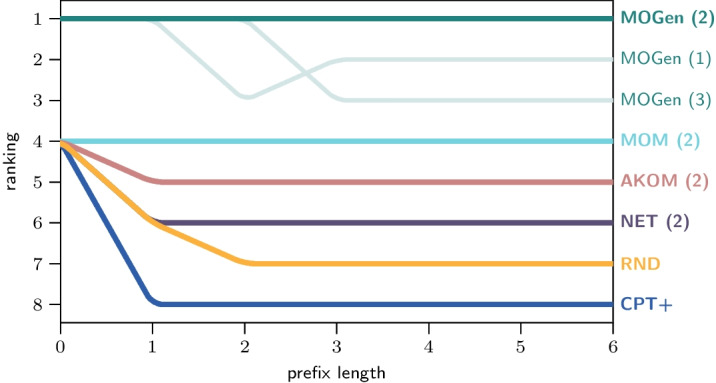
Prediction performance on different prefix lengths for MSNBC

To better understand where the prediction performance originates, we provide additional results comparing the performance separately for each individual prefix lengths *n*. In Fig. 2, we show a ranking of methods for the different prefix lengths used for the MSBNC data set. For MOGen, we show the performance of models with maximum order *K* between 1 and 3. The best performing model ($K=2$, printed in bold) is the one identified as most suitable by our AIC-based model selection discussed below. We report the model parametrisation results for the remaining methods that yield the best performance (shown in brackets). MOGen outperforms the other methods not only across all prefix lengths, but also for each prefix length individually.

### Selection of optimal maximum order from data

Second, we assess MOGen’s model selection approach (see the Materials and Methods section). The aim of model selection is to identify the model that provides the optimal balance between model complexity and explanatory power, allowing such a model to generalise to unseen data. Existing works use either heuristic techniques (Xu et al. 2016; Rosvall et al. 2014; Pitkow and Pirolli 1999; Gueniche et al. 2015) or analytical methods that require additional model characteristics like, e.g., a nested structure or convergence properties (Scholtes 2017; Casiraghi 2021). MOGen’s model selection approach, instead, avoids computationally expensive parameter search algorithms that are used by competing methods.

In the case of MOGen, the only free parameter is the maximum order *K* of the multi-order transition matrix. Theorem 1 provides an AIC-based model selection approach that enables us to estimate the optimal value ${\hat{K}}$ of this free parameter directly from the data. Using this approach, MOGen balances the *explanatory power* of the resulting model with its *complexity*, captured by the degrees of freedom.

In Table 2b, we report the output of this model selection for the six empirical data set (top row), comparing it to the maximum order *K* that yields the best prediction performance (bottom row). For the clickstream data BMS1, FIFA, and WIKI, we obtain an optimal maximum order of $K=1$. For MSNBC, AIR, and TUBE, we obtain optimal values of $K=2$ and $K=6$, respectively, highlighting the need for higher-order models. For all analysed data sets, the optimal order detected by the model selection provides the best prediction performance in cross-validation. With this, we confirm that MOGen yields models neither underfitting nor overfitting sequential patterns in paths, effectively making it parameter-free.

### Out-of-sample prediction of paths

Third, we assess MOGen’s ability to predict full variable-length paths. Existing works have focused on modelling patterns in the sequence of traversed nodes, rather than *generative models* allowing the out-of-sample prediction of paths (Lambiotte et al. 2019). MOGen constrains only those transition sequences for which the underlying path data provides sufficient statistical evidence. The model allows all other transitions, enabling us to generate new *unseen* paths. In other words, given a set of paths, MOGen allows us to predict which other paths are likely to be observed and at which frequency.

Path prediction opens new opportunities and applications, e.g., in the management of travel disruptions, product recommendation, or online purchase prediction. We show that MOGen allows for the accurate out-of-sample prediction of full variable-length paths, even when trained on small samples of observations.

We evaluate MOGen’s performance in an out-of-sample path prediction task for the following scenario. We aim to predict passenger itineraries in the London Tube based on a training set consisting of 1000 observed paths as we would obtain, e.g., from a survey study. Based on the training set, we fit a MOGen with $K=6$, which we then use to generate a new set $S'$ of 100,000 paths of variable lengths. We rank paths in the generated set $S'$ based on their frequencies and use the top-*N* paths to predict the top $10 \%$ most frequent paths in the validation set. We interpret this prediction as a binary classification and repeat the prediction for different discrimination thresholds *N*. We then compute true-positive/false-positive rates for different values of *N* and compute the area under a ROC (receiver operating characteristic) curve.

**Fig. 3:**
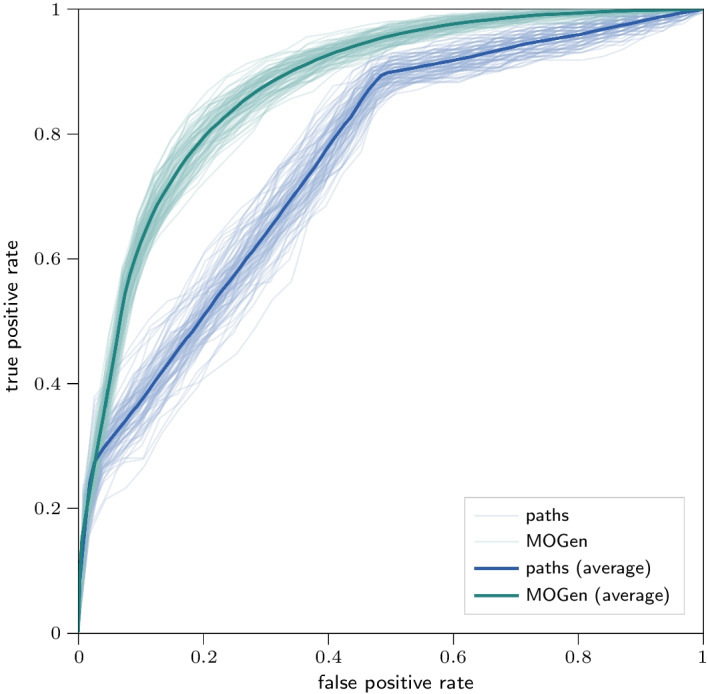
Out-of-sample path prediction with MOGen. ROC for 1000 training and 4,294,731 validation paths for TUBE

In Fig. 3, we show ROC curves (and averages) for the out-of-sample path prediction experiment repeated for 100 train-validation splits. We report prediction results for MOGen and a model-less baseline based directly on the training paths. The baseline assumes that the path frequencies in the validation data are identical to the path frequencies in the training data. These two frequencies converge as the size of the training set grows. To make the baseline predictions, we resample 100,000 paths from the training set according to the observed frequencies.

Predicting the top-*N* paths using MOGenyields an AUC of $0.87\pm 0.01$. In contrast, predicting directly from the training set based on their observed frequencies results in a significantly lower average AUC score with a higher standard deviation ($0.76\pm 0.02$). The ROC curve for the baseline contains linear segments because most paths are observed only once or twice, i.e., the training path counts alone do not provide enough information to compute relative rankings between them. MOGen is able to recover the information about paths’ probabilities from transition- and subpath-frequencies observed in the training data resulting in the higher AUC scores seen in Fig. 3. Thus, using MOGen yields a *better* and *more consistent* prediction.

In an additional experiment, we compare the path frequencies generated from MOGen and the baseline against the ‘true’ path frequencies observed in the validation set. We do so by computing Kendall’s $\tau$ rank correlation coefficients for the two generated path sets against the validation set. Kendall’s $\tau$ correlation measures the similarity of the rankings obtained by ordering the paths according to the generated frequencies with the ranking obtained according to the ‘true’ frequencies in the validation data. The average baseline’s correlation coefficient is $0.11\pm 0.02$. A low correlation is expected as the training set contains only 1000 out of over 4 million total paths. Notably, the correlation based on MOGen’s generated paths is significantly higher, averaging $0.40\pm 0.01$. This result shows that the new paths generated by MOGen and their distribution are compatible with the validation data. Thus, the remarkable improvement in predictive performance originates from MOGen generating new paths not present in the training data but compatible with the observed transitions and the underlying network topology.

### Scalability

Fourth, we show that MOGen is highly scalable to large sets of path data both in terms of time and memory requirements. The scalability results from the modular multi-layer formulation of the method (see the Materials and Methods section), which allows for an efficient sparse representation and a parallel implementation.

**Fig. 4:**
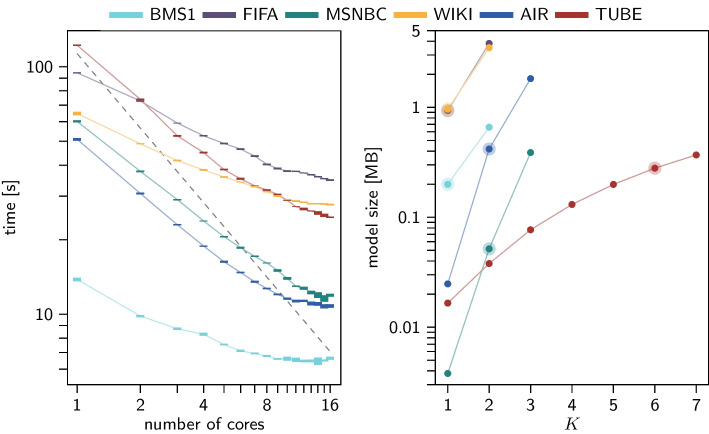
The left panel shows the mean and standard deviations of the times required for training model selection (five iterations). The grey line in the left panel indicates a linear speedup. The right panel shows the resulting model size for all datasets. Highlighted models in the right panel are the ones picked through model selection

Our implementation parallelises the training of a multi-order model by splitting paths into multiple sets and simultaneously computing entries of the multi-order adjacency matrix ${{\textbf {A}}}^{(K)}$ and transition matrix ${{\textbf {T}}}^{(K)}$ for each of the sets, and finally aggregating those entries. We speed up the computation of the model likelihood (cf. Eq. 4) by computing the probabilities of paths in parallel. The left panel of Fig. 4 shows the time (in seconds) that is required to (i) train multi-order models for multiple parameters *K*, and (ii) select the optimal parameter ${\hat{K}}$ using the method described in Eq. 2 for different numbers of processing cores.^3^ The resulting model sizes (in MegaBytes) for different orders *K* are shown in the right panel of Fig. 4.

The results show that the time required to fit an optimal model primarily depends on the number of unique paths as well as their length (cf. Table 1). We also observe a near-linear reduction of the training time as we increase the number of processing cores (grey line refers to an optimal linear speedup). The deviation from the linear reduction originates from the non-parallel implementation of the matrix powers of ${\varvec{\mathcal {A}}}$, required to compute the degrees of freedom. The size of the models generally increases super-linearly with the maximum order *K*. The scaling of model size depends on the size and algebraic connectivity of the underlying topology, which determines the growth of the degrees of freedom with the parameter *K*. This is illustrated by the TUBE data set, where the increase in model size is lowest as the order increases due to a sparsely connected network topology. All models selected by MOGen are below 1 MB in size.

## Discussion

Path data, i.e., time-ordered ordered sequences of nodes that are traversed, e.g., by passengers in a transportation network, users navigating in a network of hyperlinked articles, or processes propagating in a complex network are increasingly available in a wide range of scientific disciplines. Modelling and understanding sequential patterns in such data, especially those that cannot be explained by the underlying network’s topology, promises to fundamentally advance our understanding of complex systems. Such patterns not only capture how the elements of a system indirectly influence each other, they also enable us to *predict* paths in networks, with applications, e.g., in the design of recommender systems, smart mobility services, or eCommerce. To do so, we must *simultaneously* account for three characteristics of real-world path data: (i) we typically have large collections of (short) paths with variable lengths, (ii) each of those paths consists of a time-ordered node sequence, and (iii) possible node sequences are constrained by an underlying network topology. Despite their importance, the modelling of sequential patterns in such data remains an open challenge as state-of-the art methods, at most, account for two of those characteristics at the same time.

Closing this gap, we introduced MOGen, a generative modelling framework to learn compact higher-order models of sequential patterns in paths that account for both the temporal ordering and variable-length of paths, as well as underlying network constraints. Addressing the challenge of overfitting, MOGen provides an integrated model selection that yields compact and generalisable graphical models for patterns in variable-length paths. Our results confirmed that the selected models are indeed those that generalise best to unseen data, effectively making our method parameter-free. Using empirical data, we showed that MOGen enables both next-element prediction and out-of-sample prediction of full variable-length paths with high accuracy and consistency. Moreover, it outperforms state-of-the-art sequence modelling algorithms and higher-order network models when predicting the next visited element, as we showed for six empirical path data sets. We finally showed that we can use it to generate *surrogate data*, i.e., new unseen paths that are compatible with patterns observed in the data. MOGen’s formalisation allows for a highly efficient and scalable representation as a sparse multi-order transition matrix. By simultaneously modelling sequential and topological patterns in empirical data on paths in networks, the resulting models further advance our understanding of complex systems. We provide a parallel implementation of MOGen as an Open Source project.

In future work, we will further evaluate the novel opportunities that path generation and multi-order network analysis with MOGen open in real-world applications. We will further explore the link between MOGen and related methods from the domain of language modelling.

## Materials and methods

### MOGen formalisation

We first formally define path data and present the generative modelling framework and model selection algorithm that are the main contributions of our work. We assume that we are given a multi-set of *m* paths $S = \{t_1, \dots , t_m\}$ on a network $G = (V, E)$ with nodes *V* and (directed) links $E \subseteq V \times V$. Each path $t_i = v_1 \rightarrow v_2 \rightarrow \dots \rightarrow v_{l_i}$ is an ordered sequence of traversed nodes under the constraint that $(v_{j}, v_{j+1}) \in E$ for $j \in [1, l_i-1]$. We assume that paths in *S* have varying lengths $l_i$, and we denote the maximum path length as $l_{max}$. If we were to use a simple network-based model for the sequences of nodes traversed by paths, we would assume that paths were generated by a memoryless random walk corresponding to a first-order Markov chain. The nodes $v \in V$ correspond to the state space of such a Markov chain, while links $(v,w) \in E$ are associated with transition probabilities capturing the probability of the transition from *v* to *w*. This assumes that the next node $v_{i+1}$ traversed by a path only depends on (i) the currently visited node $v_{i}$, and (ii) the links that determine which state transitions are possible. To capture higher-order dependencies in the sequence of traversed nodes that are not due to the network topology, we can generalise this model to a *k*-th order Markov chain, where the next node $v_{i+i}$ depends on the *k* previously visited nodes $v_{i-k},..., v_{i}$. We can view this model as a memoryless random walk in a *k*-th order network $G^{k}=(V^{k}, E^{k})$, where (i) each *k*-th order node $(v_1,..., v_k) \in V^k$ is a sequence of *k* nodes where consecutive nodes are connected by links, and (ii) each *k*-th order link connects two higher-order nodes $(v_1,..., v_k)$ and $(w_1,..., w_k)$ such that $w_{i}=v_{i-1} \in V$.

#### Multi-order models of paths

The modelling approach above has several limitations with respect to the unique characteristics of variable-length paths in *S*. (i) Using a *k*-th order model, we can neither use the first *k* nodes in each path to fit the transition probabilities of the model nor can we use the model to perform a next-element prediction for the first *k* nodes on a path. (ii) The fact that the number of observations used to fit the transition probabilities of a *k*-th order model differs across different orders *k* complicates model selection, i.e., the selection of the optimal order *k* to model a data set. (iii) The model neglects information on the start- and endpoint of paths, hindering its application to an out-of-sample prediction of full variable-length paths, i.e., from start to end. (iv) Finally, a model with a single order *k* is limited to capture sequential patterns of a single length *k*, which is why recent works argued for variable- and multi-order modelling techniques (Scholtes 2017; Xu et al. 2016; Pitkow and Pirolli 1999).

To overcome those limitations, we introduce a generative modelling framework that combines (i) transitions between higher-order models of multiple orders up to a maximum order *K* and (ii) special transitions that capture the start and endpoints of paths. For this, we introduce a special *initial state* $*$, with transitions $* \rightarrow v$ to first-order nodes $v \in V$ that mark the start of a path in node *v*. For all orders $1 \le k \le K$, we further add transitions from *k*-th order nodes $(v_1,..., v_k) \rightarrow \dagger$ to a special *terminal state* $\dagger$, which captures the termination of a path. With this extension, a path $v_1 \rightarrow v_2 \rightarrow \dots \rightarrow v_{l}$ corresponds to $l+2$ transitions in a *multi-order network* with maximum order *K*, which gives rise to the following sequence of *l* higher-order nodes

$$\begin{aligned} * \rightarrow v_1 \rightarrow (v_1, v_2) \rightarrow \dots \rightarrow (v_{l-K+1},..., v_{l}) \rightarrow \dagger \end{aligned}$$

with two additional transitions from/to the special states $*$ and $\dagger$ to model the start and end of the path. Considering the coding of memory prefixes in the extended state space of higher-order Markov chains, we note that each transition in this multi-order model with maximum order *K* increases the memory prefix by one node, up to a maximum memory of length *K*. In addition to transitions *within* a *K*-th order model, we obtain a multi-order model that includes transitions between the nodes of a *k*-th order model and the nodes of a $k+1$-th order model for all orders $k<K$. Transitions from/to the initial state $*$ and terminal state $\dagger$ explicitly model the start- and endpoints of paths, which is the basis for an end-to-end, out-of-sample prediction of full paths.

**Fig. 5:**
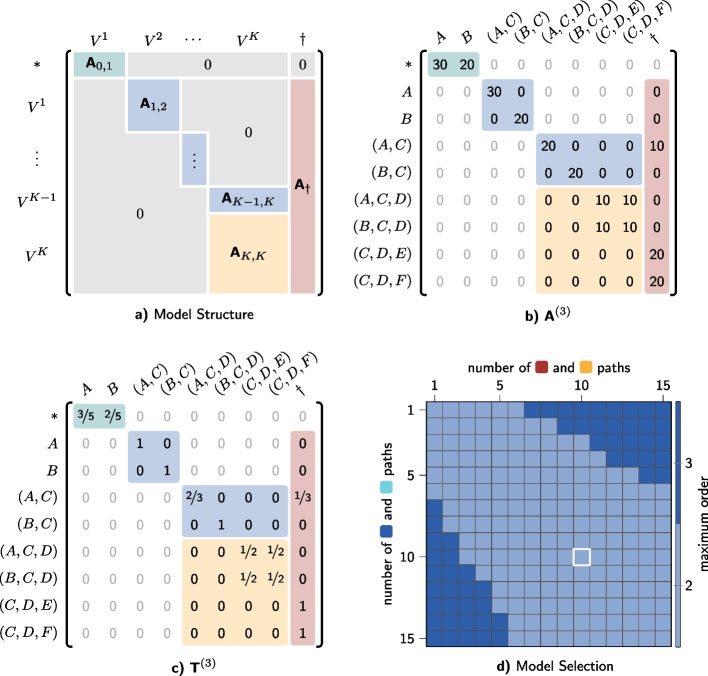
Multi-order matrix representation of MOGen. **a** shows the block structure of the multi-order adjacency matrix. **b** and **c** show ${{\textbf {A}}}$ and ${{\textbf {T}}}$ for MOGen with $K=3$ on the example from Fig. 1a, where all paths occur 10 times. **d** shows the *K* detected by model selection for varying observation counts. The observation from (**b**) and (**c**) is highlighted in white

We can mathematically represent such a multi-order network with maximum order *K* through a *multi-order adjacency matrix* ${{\textbf {A}}}^{(K)}$, whose overall structure is shown in Fig. 5a. This representation is inspired by so-called *supra-adjacency matrices*, which have recently been proposed to represent the interconnected topologies of multi-layer networks (Kivelä et al. 2014). A multi-order adjacency matrix consists of blocks ${\textbf{A}}_{k-1,k}$, whose entries count the observed transitions between $k-1$-th and *k*-th order nodes in a given multi-set *S* of paths. The entries in the top-left block ${{\textbf {A}}}_{0,1} \in {\textbf{R}}^{1\times n}$ count transitions $(*, v)$ for all nodes $v \in V$, i.e., the distribution of start nodes for all paths. The entries of the bottom-right block ${\textbf{A}}^{\dagger }$ count *terminal transitions* $(v_{1},..., v_{k}) \rightarrow \dagger$ for all orders $1 \le k \le K$. For paths with lengths $l_i>K$, the entries in the bottom matrix ${\textbf{A}}_{K,K}$ count the transitions between the nodes in a *K*-th order network model. Figure 5a shows an example for a multi-order adjacency matrix that corresponds to a toy example where each path shown in Fig. 1a is observed ten times. We note that structural zeros in ${{\textbf {A}}}^{(K)}$ (greyed out in Fig. 5b) represent transitions that are *impossible* based on the underlying network topology, which are different from zero entries that represent possible but *unobserved* transitions (see zeros in highlighted blocks in  Fig. 5b). Considering these structural zeros allows for a sparse memory-efficient representation of our method. The only model parameter *K* determines the maximum order of the multi-order model, which resembles the maximum memory length in a Markov chain. We note that, under the assumption that the paths in a multi set $S=\{ t_1, \ldots , t_m\}$ with maximum path length $l_{max}$ are generated independently of each other, the multi-order adjacency matrix ${{\textbf {A}}}^{(K)}$ is a lossless representation of *S* iff $K \ge l_{max}$. To facilitate prediction tasks, we use the multi-order adjacency matrix to define a *probabilistic generative model* of variable-length paths. This model is fully described by a *multi-order transition matrix* ${{\textbf {T}}}^{(K)}$, which we obtain by a row normalisation of ${{\textbf {A}}}^{(K)}$. This yields a stochastic matrix ${{\textbf {T}}}^{(K)}$, whose entries give the transition probabilities in a probabilistic model for paths.

The transition matrix of a generative multi-order model with maximum order $K=3$ that corresponds to the toy example in Fig. 1a is shown in Fig. 5c. To illustrate our method in the toy example shown in Fig. 1a, we present here results for a simple experiment. Varying the observed frequencies of the five (colour-coded) paths shown in Fig. 1a, we report the different estimated optimal orders ${\hat{K}}$. Due to the topology of the network shown in Fig. 1a, paths that start in node *A* or *B* and continue to *C* and *D* either terminate in node *E* or node *F*. In order to model paths whose probability to terminate in *E* or *F* depends on whether they start in *A* or *B*, a MOGen with maximum order three is needed. Such a dependency exists if the paths $A \rightarrow C \rightarrow D \rightarrow E$ and $B \rightarrow C \rightarrow D \rightarrow F$ occur more or less frequently than the paths $B \rightarrow C \rightarrow D \rightarrow E$ and $A \rightarrow C \rightarrow D \rightarrow F$. Figure 5d shows that our model selection algorithm yields the correct optimal order. If, however, the terminal node of a path is independent of the start node, a model with order two is sufficient. This is the case if the frequencies of all four paths are similar, in which case our model selection algorithm yields the correct value for ${{\hat{K}}}=2$ (cf. Figure 5d). This is illustrated by the identical values in ${{\textbf {T}}}^{(3)}_{3,3}$ in Fig. 5c. These results merely illustrate our method. We perform a thorough cross-validation experiment in the Results section. With this, we confirm that the best prediction performance is obtained for the parameter ${\hat{K}}$ estimated according to Theorem 1, which we introduce in the next section. This implies that, different from existing techniques, the parameter *K* can be directly estimated from the data, which effectively turns our method into a *parameter-free approach*.

**Optimal maximum order detection**

The unique formulation of MOGen allows the comparison of likelihoods across different maximum orders *K*. Thanks to this, we can utilise Akaike’s Information Criterion (AIC) (Akaike 1974) to choose MOGen’s optimal maximum order ${{\hat{K}}}$. AIC balances the *explanatory power* of a model, given by its likelihood function $\mathcal {L}({{\textbf {T}}}^{(K)}\vert S)$, with its *complexity*, measured in terms of the model’s degrees of freedoms $d({\textbf{T}}^{(K)})$. Given a multi set *S* of *m* independent paths, $\mathcal {L}({\textbf{T}}^{(K)}\vert S)$ is simply the product of all path probabilities ${P}(t\vert {{\textbf {T}}}^{(K)})$. The probability ${P}(t\vert {{\textbf {T}}}^{(K)})$ of a single observed path *t* in *S* is instead given by the probability of observing all its transitions in sequence, as specified by ${\textbf{T}}^{(K)}$. This formalisation is easily parallelisable. Therefore, as we show in our results, MOGen is highly scalable and memory efficient.

The degrees of freedom $d({{\textbf {T}}}^{(K)})$ need to account for (i) structural zeros in $T^{(K)}$ resulting from paths being restricted to a network, (ii) the multi-layer structure of MOGen combining multiple orders *k*, and (iii) additional model parameters used to model transitions from/to the initial and terminal states. Thus, $d({{\textbf {T}}}^{(K)})$ depends on the topology of the underlying network,^4^ encoded by ${\varvec{\mathcal {A}}}$. Possible paths of length *k* in such a network are encoded by the non-zero entries of ${\varvec{\mathcal {A}}}^k$. These specify which transition probabilities need to be estimated from the data *S* and which are structural zeros. The degrees of freedom of a MOGen/ with maximum order *K* are then:

1 $$\begin{aligned} d({{\textbf {T}}}^{(K)}) = \sum _{k=1}^K \sum _{i,j}\left( {\varvec{\mathcal {A}}}^{k}\right) _{ij} + \vert V\vert - 1 \end{aligned}$$

Since the best model among a set of candidate models is the one that minimises the AIC, we can compute the optimal maximum order of a MOGen model given a set of paths as follows:

##### Theorem 1

Let $S=\{t_i\}$ be a multi-set consisting of *m* independent paths. Let ${\textbf{T}}^{(K)}$ be the multi-order transition matrix defining the MOGen of maximum order *K* constructed from *S*. Let ${\varvec{\mathcal {A}}}$ be the binary adjacency matrix where ${\varvec{\mathcal {A}}}_{vw}=1$ iff the transition $v\rightarrow w$ appears at least once in *S*. Then the optimal maximum order ${\hat{K}}$ according to AIC is

2 $$\begin{aligned} {\hat{K}} := {\text {arg min}}_{K} \sum _{k=1}^K \sum _{i,j}\left( {\varvec{\mathcal {A}}}^{k}\right) _{ij} - \sum _{t\in S}\ln {P}(t\vert {{\textbf {T}}}^{(K)}), \end{aligned}$$

where ${P}(t\vert {{\textbf {T}}}^{(K)})$ denotes the probability of a path $t\in S$.

##### **Formal Proof of Theorem**

1 We split the formal proof of Theorem 1 in a series of small lemmas. Doing so, we go through all the steps needed to obtain Eq. 2. The first step consists of specifying the probability of observing a path *t* according to a multi-order model defined by a multi-order transition matrix ${\textbf{T}}^{(K)}$.

##### Lemma 1

(Probability of a path) Let ${\textbf{T}}^{(K)}$ be a multi-order transition matrix defining a multi-order model of maximum order *K*. Then, the probability ${P}(t\vert {\textbf{T}}^{(K)})$ according to ${\textbf{T}}^{(K)}$ of observing a path $t=v_1 \rightarrow \dots \rightarrow v_l$ is

3 $$\begin{aligned} \begin{aligned} {P}(t\vert {{\textbf {T}}}^{(K)}) =&~{{\textbf {T}}}^{(K)}[*,v_1] \cdot \prod _{k=1}^{K-1} {{\textbf {T}}}^{(K)}[({v_1,..., v_k}), (v_1,..., v_{k+1})] \cdot \\&\prod _{i=K+1}^{l} \hspace{-1.3mm} {{\textbf {T}}}^{(K)}[(v_{i-K},..., v_{i-1}), (v_{i-K+1},..., v_{i})] \cdot \\&{{\textbf {T}}}^{(K)}[(v_{l-K+1},..., v_l), \dagger ]. \end{aligned} \end{aligned}$$

##### Proof

In the multi-order representation, a path $t=v_1 \rightarrow \dots \rightarrow v_l$ corresponds to $l+2$ transitions along the edges of a multi-order network with maximum order *K*, which gives rise to the following sequence of *l* higher-order nodes:

$$\begin{aligned} * \rightarrow v_1 \rightarrow (v_1, v_2) \rightarrow \dots \rightarrow (v_{l-K+1},..., v_{l}) \rightarrow \dagger \end{aligned}$$

The probability of each of these transitions is (a) independent by definition and (b) completely specified by the multi-order transition matrix ${\textbf{T}}^{(K)}$. Thus, the probability ${P}(t\vert {{\textbf {T}}}^{(K)})$ of observing a path *t* is given by the product of the $l+2$ probabilities given in ${\textbf{T}}^{(K)}$ of observing each higher-order transition defining the path. $\square$

Employing the results of Lemma 1, we can compute the probability of a multi-set *S* of independent paths.

##### Lemma 2

(Probability of a paths’ collection) Let ${\textbf{T}}^{(K)}$ be a multi-order transition matrix defining a multi-order model of maximum order *K*. Let $S=\{t_i\}$ be a multi-set consisting of *m* independent paths. Then, the probability of observing *S* according to ${\textbf{T}}^{(K)}$ is

4 $$\begin{aligned} {P}(S\vert {{\textbf {T}}}^{(K)}) = \prod _{t \in S}{P}(t\vert {{\textbf {T}}}^{(K)}), \end{aligned}$$

where ${P}(t\vert {{\textbf {T}}}^{(K)})$ is given by Eq. 3.

##### Proof

All paths *t* in *S* are assumed to be independent. Thus, the probability ${\textbf{T}}^{(K)}$ of observing *S* according to ${\textbf{T}}^{(K)}$ is simply the product of the probabilities ${P}(t\vert {{\textbf {T}}}^{(K)})$ of observing each path $t\in S$. $\square$

These first two lemmas allow computing the likelihood of a multi-order model given some observed data in the form of a (multi-)set of paths. The final lemma allows defining the number of degrees of freedom of a multi-order model constrained by a graph *G*(*V*, *E*).

##### Lemma 3

(Number of possible transitions) Let ${\varvec{\mathcal {A}}}$ be the first-order binary adjacency matrix specifying a graph *G*(*V*, *E*) over which a multi-order model ${{\textbf {T}}}^{(K)}$ of maximum order *K* is defined. Then, the maximum number $N_\tau$ of possible transitions in ${{\textbf {T}}}^{(K)}$ is given by

5 $$\begin{aligned} N_\tau = \vert V\vert + \sum _{k=1}^K \sum _{i,j}\left( {\varvec{\mathcal {A}}}^{k}\right) _{ij} + \vert \bigcup _{k=1}^{K} V^k\vert , \end{aligned}$$

where $V^k$ denotes the set of connected *k*-th order nodes in ${{\textbf {T}}}^{(K)}$.

##### Proof

Because the multi-order model is defined over a graph *G*(*V*, *E*), to all multi-order transition probabilities in ${{\textbf {T}}}^{(K)}$ correspond first-order edges in *E*. Similarly, for all edges $e\in E$, there is at least one non-zero multi-order transition probability in ${{\textbf {T}}}^{(K)}$ that encompasses *e*. Thus, $\sum _{k=1}^K \sum _{i,j}\left( {\varvec{\mathcal {A}}}^{k}\right) _{ij}$ gives the number of all transitions up to order *K* that are possible according to *G*(*V*, *E*). Furthermore, there possibly exist $\vert V\vert$ transitions from the initial state $*$ to any node in *V*. Finally, there possibly exist $\vert \bigcup _{k=1}^{K} V^k\vert$ transitions from any higher-order node in $\bigcup _{k=1}^{K} V^k$ to the terminal state $\dagger$. Summing over all possible transitions, we get to Eq. 5. $\square$

With the results provided by the above lemmas, we can proceed with a proof for Theorem 1.

##### Proof of of Theorem 1

For a multi-order model, we can calculate the AIC as follows:

6 $$\begin{aligned} \text {AIC}({{\textbf {T}}}^{(K)}) = 2d({{\textbf {T}}}^{(K)}) - 2\ln \left\{ \mathcal {L}({{\textbf {T}}}^{(K)}\vert S)\right\} , \end{aligned}$$

where $d({{\textbf {T}}}^{(K)})$ denotes the number of degrees of freedom of the model identified by ${{\textbf {T}}}^{(K)}$, and $\mathcal {L}({{\textbf {T}}}^{(K)}\vert S)$ its likelihood given the data *S*.

The number of degrees of freedom $d({{\textbf {T}}}^{(K)})$ of a multi-order model corresponds to the number of multi-order transition probabilities in ${{\textbf {T}}}^{(K)}$ that have to be estimated from the data. The elements of ${{\textbf {T}}}^{(K)}$ can be divided into two classes: structural zeros and possible transitions. Structural zeros are those multi-order transitions that cannot be observed, and thus their probabilities are set to zero and do not contribute to the number of degrees of freedom of the model. The values of the remaining transition probabilities have to be estimated from the data. Thus, they contribute to the degrees of freedom of the model. For this reason, $d({{\textbf {T}}}^{(K)})$ can be computed by counting how many transitions are possible according to the observed data *S*. We denote with ${\varvec{\mathcal {A}}}$ the binary adjacency matrix whose elements ${\varvec{\mathcal {A}}}_{vw}=1$ iff a transition $v\rightarrow w$ has been observed at least once in *S*. Given ${\varvec{\mathcal {A}}}$, we can find the maximum number of possible transitions $N_\tau$ using Lemma 3. Furthermore, we note that the multi-order transition matrix ${{\textbf {T}}}^{(K)}$ is row-stochastic. The number of degrees of freedom $d({{\textbf {T}}}^{(K)})$ is then given by $N_\tau$ minus the number of rows $r = \vert \bigcup _{k=1}^{K} V^k\vert + 1$ of ${{\textbf {T}}}^{(K)}$. The reason for this is that by accounting for the row-stochasticity of ${{\textbf {T}}}^{(K)}$ one transition probability *per row* need not be estimated:

7 $$\begin{aligned} \begin{aligned} d({{\textbf {T}}}^{(K)})&= N_\tau - r \\&= \vert V\vert + \sum _{k=1}^K \sum _{i,j}\left( {\varvec{\mathcal {A}}}^{k}\right) _{ij} + \vert \bigcup _{k=1}^{K} V^k\vert - \vert \bigcup _{k=1}^{K} V^k\vert - 1 \\&= \vert V\vert + \sum _{k=1}^K \sum _{i,j}\left( {\varvec{\mathcal {A}}}^{k}\right) _{ij} - 1. \end{aligned} \end{aligned}$$

This provides us with an explicit expression for the first term in Eq. 6.

To get to Eq. 2, we further need to compute the likelihood $\mathcal {L}({{\textbf {T}}}^{(K)}\vert S)$ of the model defined by ${{\textbf {T}}}^{(K)}$. As $\mathcal {L}({{\textbf {T}}}^{(K)}\vert S) = {P}(S\vert {{\textbf {T}}}^{(K)})$, we can use Theorem 1,2 to express it in terms of the probabilities ${P}(t\vert {{\textbf {T}}}^{(K)})$ of the paths $t\in S$.

Finally, we highlight that the best model among a set of candidate models is the one that minimises the AIC. Thus, plugging Eq. 7,4 in Eq. 6 and taking the argmin of the resulting expression gives us Eq. 2 (after ignoring constant terms), concluding this proof. $\square$

### Experimental setup for next-element prediction

#### Selection of baseline models

We base our selection of baseline methods for next-element prediction on the recent review (Tax et al. 2020). The authors of Tax et al. (2020) find that AKOM (Pitkow and Pirolli 1999) outperforms all other algorithms in three out of four tested data sets. Conversely, in Gueniche et al. (2015) the authors of CPT+ show that their algorithm outperforms AKOM in terms of the accuracy of the next element prediction. We thus use both CPT+ and AKOM as baselines, utilising the implementations provided by the authors of Fournier-Viger et al. (2014). To enable a fair comparison for empty prefixes, we modified the implementation of AKOM to return a prediction based on the frequency of elements in the training data. This is described in Pitkow and Pirolli (1999). However, the implementation in Fournier-Viger et al. (2014) returns an empty prediction instead. CPT+ predicts the next element in a sequence based on an internal assignment of weights to candidate elements, i.e., the prediction is not based on probabilities. We thus normalise the weights assigned by CPT+ to obtain a probabilistic prediction. While this allows us to compare the performance of CPT+ with the other (probabilistic) methods, we highlight that the evaluation in Gueniche et al. (2015) is based on prediction accuracy. It is crucial noting that the authors of Tax et al. (2020) report that AKOM and state-of-the-art RNNs achieved the highest and, at the same time, very similar levels of performance on four datasets. As our method is based on a Markov chain model, we thus opt to use the comparable method AKOM as a benchmark for our method and not RNNs. Based on the findings in Tax et al. (2020), we expect these results to hold also for RNNs.

In addition to those sequence modelling techniques, we compare our method to a probabilistic prediction derived from (i) a random walk in a higher-order network with a single order *k* (NET), and (ii) the multi-order graphical model (MOM) introduced in Scholtes (2017). For $k=1$, NET is identical to a prediction generated by a random walk in the underlying network. Note that, despite the similar name, the method proposed in the present work considerably differs from MOM. First, MOM does not model the endpoint of paths, limiting its ability to predict variable-length paths. Second, it uses a model-fitting approach that estimates transition probabilities based on sub-path frequencies rather than the transition probabilities used in our approach. In addition to those network-based models, we use a naive (parameter-free) random baseline (RND) that simply predicts the next node based on the relative frequency of nodes in the training set, i.e., a prediction that neither uses a Markov chain nor the network topology.

#### Selection of model parameters

AKOM, MOM, NET, and MOGen are characterised by a single parameter, i.e., their (maximum) order. We search for the optimal order in terms of predictive performance. We obtain this optimal order by iteratively increasing the model order from one until a maximum in predictive performance is reached. For CPT+, we use the parameters proposed in Gueniche et al. (2015). Our naive random baseline (RND) is parameter-free. We report the optimal (maximum) orders for all models and data sets in Table 3.

**Table 3:** Best performing model parameters for all data sets

	BMS1	FIFA	MSNBC	WIKI	AIR	TUBE
MOGen	1	1	2	1	2	6
AKOM	1	1	2	1	2	6
NET	1	1	2	1	1	1
MOM	1	1	2	1	3	6

#### Cross-entropy loss

Following (Bishop and Nasrabadi 2006), we define the cross-entropy loss function for next-element prediction as

8 $$\begin{aligned} H(p,q) := - \sum _{v \in V} p(v) \log q(v) \end{aligned}$$

where for a given prefix $(v_1,..., v_k)$, *q*(*v*) denotes the probability of the next element (i.e., target node) *v* obtained from a model trained on the training set and *p*(*v*) is the true distribution underlying the data. Since the true distribution is unknown for the empirical data set, we compute *p*(*v*) from the distribution of actual next nodes in the validation set. This yields the log-loss function commonly used in the cross-validation of probabilistic multi-class prediction (Bishop and Nasrabadi 2006). We evaluate predictions for multiple prefix lengths up to a maximum length of six. Those are considered as multiple samples and assign equal sample weights such that sample weights for any given target node with different prefix lengths sum to one. For the resulting loss function, a value of zero corresponds to a perfect prediction, which would allow us to replace the true distribution in the data with the prediction without information loss. Non-zero values quantify the information loss in bits, i.e., larger values correspond to worse prediction performance.

## Data Availability

All datasets used in this study are publicly available at sources cited in Table 1. We provide an Open Source parallel implementation of MOGen integrated in https://github.com/pathpy/pathpy. We have further prepared a full reproducibility package which we will publish on Zenodo upon acceptance.

## Footnotes

1 Note that we do not assume that nodes or edges are unique, i.e., we do not distinguish between paths, trails, or walks (Bollobás 1998).

2 We obtained the data sets BMS1, FIFA and MSNBC from Fournier-Viger et al. (2014). All other data sets were obtained from the cited publications.

3 The results were obtained using a 16-core Intel Core i9-7960X processor, 128 GB of memory, and Ubuntu 18.04.

4 If we assume that all possible transitions along the links of the underlying network have been observed at least once, we can directly derive ${\varvec{\mathcal {A}}}$ from the training data.

## References

[CR1] Akaike H (1974). A new look at the statistical model identification. IEEE Trans Autom Control.

[CR2] Arlitt M, Jin T (2000). A workload characterization study of the 1998 world cup web site. IEEE Network.

[CR3] Augusto A, Conforti R, Dumas M, La Rosa M (2017) Split miner: discovering accurate and simple business process models from event logs. In: 2017 IEEE international conference on data mining (ICDM). IEEE, pp 1–10

[CR4] Balle B, Carreras X, Luque FM, Quattoni A (2014). Spectral learning of weighted automata. Mach Learn.

[CR5] Belth C, Kamran F, Tjandra D, Koutra D (2019) When to remember where you came from: node representation learning in higher-order networks. In: Proceedings of the 2019 IEEE/ACM international conference on advances in social networks analysis and mining, pp 222–225

[CR6] Benson AR, Gleich DF, Higham DJ (2021) Higher-order network analysis takes off, fueled by old ideas and new data. SIAM News. https://cutt.ly/gkwhM9w. Last accessed 29 Jan

[CR7] Benson AR, Gleich DF, Lim L-H (2017). The spacey random walk: a stochastic process for higher-order data. SIAM Rev.

[CR8] Bernhard SD, Leung CK, Reimer VJ, Westlake J (2016) Clickstream prediction using sequential stream mining techniques with markov chains. In: Proceedings of the 20th international database engineering & applications symposium, pp 24–33

[CR9] Bestavros A (1995) Using speculation to reduce server load and service time on the www. In: Proceedings of the fourth international conference on information and knowledge management, pp 403–410

[CR10] Bishop CM, Nasrabadi NM (2006). Pattern recognition and machine learning.

[CR11] Bollen J, Sompel H, Hagberg A, Bettencourt L, Chute R, Rodriguez MA, Balakireva L (2009). Clickstream data yields high-resolution maps of science. PLoS ONE.

[CR12] Bollobás B (1998). Modern graph theory.

[CR13] Brockmann D, Helbing D (2013). The hidden geometry of complex network-driven contagion. Phenomena Sci.

[CR14] Brodley C, Kohavi R (2000) KDD-Cup 2000 homepage. http://www.kdd.org/kdd-cup/view/kdd-cup-2000

[CR15] Buijs, JC, Van Dongen BF, Der Aalst WM (2012)On the role of fitness, precision, generalization and simplicity in process discovery. In: OTM confederated international conferences “On the Move to Meaningful Internet Systems”. Springer, pp 305–322

[CR16] Cadez I, Heckerman D, Meek C, Smyth P, White S (2000) Visualization of navigation patterns on a web site using model-based lustering. In: Proceedings of the Sixth ACM SIGKDD international conference on knowledge discovery and data mining, pp 280–284

[CR17] Casiraghi G (2021). The likelihood-ratio test for multi-edge network models. J Phys: Compl.

[CR18] Chai WK, Pavlou G (2016). Path-based epidemic spreading in networks. IEEE/ACM Trans Netw.

[CR19] Chierichetti F, Kumar R, Raghavan P, Sarlos T (2012) Are web users really Markovian? In: Proceedings of the 21st international conference on World Wide Web, pp 609–618

[CR20] Cleary J, Witten I (1984). Data compression using adaptive coding and partial string matching. IEEE Trans Commun.

[CR21] Dai W, Hu H, Wu T, Dai Y (2014) formation spread of emergency events: path searching on social networks. Sci World J 10.1155/2014/179620 PMC3926277 24600323

[CR22] Deshpande M, Karypis G (2004). Selective Markov models for predicting web page accesses. ACM Trans Internet Technol (TOIT).

[CR23] Fournier-Viger P, Gomariz A, Gueniche T, Soltani A, Wu C-W, Tseng VS (2014). Spmf: a java open-source pattern mining library. J Mach Learn Res.

[CR24] Fournier-Viger P, Lin JC-W, Kiran RU, Koh YS, Thomas R (2017). A survey of sequential pattern mining. Data Sci Pattern Recogn.

[CR25] Frias-Martinez E, Karamcheti V (2002) A prediction model for user access sequences. In: WEBKDD workshop: Web mining for usage patterns and user profiles

[CR26] Gueniche T, Fournier-Viger P, Raman R, Tseng VS (2015) Cpt+: decreasing the time/space complexity of the compact prediction tree. In: Pacific-Asia conference on knowledge discovery and data mining, pp 625–636

[CR27] Gündüz, Ş, Özsu MT(2003) A web page prediction model based on click-stream tree representation of user behavior. In: Proceedings of the ninth ACM SIGKDD international conference on knowledge discovery and data mining, pp 535–540

[CR28] Hackl J, Adey BT, Lethanh N (2018). Determination of near-optimal restoration programs for transportation networks following natural hazard events using simulated annealing. Comput-Aid Civil Infrastruct Eng.

[CR29] Hui SK, Fader PS, Bradlow ET (2009). Path data in marketing: an integrative framework and prospectus for model building. Mark Sci.

[CR30] Jo W, Chang D, You M, Ghim G-H (2021). A social network analysis of the spread of covid-19 in South Korea and policy implications. Sci Rep.

[CR31] Karlebach G, Shamir R (2008). Modelling and analysis of gene regulatory networks. Nat Rev Mol Cell Biol.

[CR32] Kim Y, Chen Y-S, Linderman K (2015). Supply network disruption and resilience: a network structural perspective. J Oper Manag.

[CR33] Kivelä M, Arenas A, Barthelemy M, Gleeson JP, Moreno Y, Porter MA (2014). Multilayer networks. J Comp Netw.

[CR34] Kraus M, Feuerriegel S (2019) Personalized purchase prediction of market baskets with wasserstein-based sequence matching. In: Proceedings of the 25th ACM SIGKDD international conference on knowledge discovery & data mining, pp 2643–2652

[CR35] Laird P, Saul R (1994). Discrete sequence prediction and its applications. Mach Learn.

[CR36] Lambiotte R, Rosvall M, Scholtes I (2019). From networks to optimal higher-order models of complex systems. Nat Phys.

[CR37] LaRock T, Nanumyan V, Scholtes I, Casiraghi G, Eliassi-Rad T, Schweitzer F (2019) HYPA: efficient detection of path anomalies in time series data on networks

[CR38] Leemans SJ, Tax N, Hofstede AH (2018) Indulpet miner: combining discovery algorithms. In: OTM confederated international conferences on the move to meaningful internet systems. Springer, pp 97–115

[CR39] Li Y, Zobel CW (2020). Exploring supply chain network resilience in the presence of the ripple effect. Int J Prod Econ.

[CR40] Montgomery AL, Li S, Srinivasan K, Liechty JC (2004). Modeling online browsing and path analysis using clickstream data. Mark Sci.

[CR41] Olson EN (2006). Gene regulatory networks in the evolution and development of the heart. Science.

[CR42] Padmanabhan VN, Mogul JC (1996). Using predictive prefetching to improve World Wide Web latency. ACM SIGCOMM Comput Commun Rev.

[CR43] Pavlov A, Ivanov D, Werner F, Dolgui A, Sokolov B (2019) Integrated detection of disruption scenarios, the ripple effect dispersal and recovery paths in supply chains. Ann Oper Res 1–23

[CR44] Peixoto TP, Rosvall M (2017). Modelling sequences and temporal networks with dynamic community structures. Nat Commun.

[CR45] Pitkow J, Pirolli P (1999) Mining longest repeating subsequences to predict World Wide Web surfing. In: UsENIX symposium on Internet technologies and systems, p 1

[CR46] RITA TransStat (2014) Origin and destination survey database. http://www.transtats.bts.gov/Tables.asp?DB_ID=125

[CR47] Rosvall M, Esquivel AV, Lancichinetti A, West JD, Lambiotte R (2014). Memory in network flows and its effects on spreading dynamics and community detection. Nat Commun.

[CR48] Saebi M, Ciampaglia GL, Kaplan LM, Chawla NV (2019) Honem: Network embedding using higher-order patterns in sequential data. arXiv:1908.05387 10.1089/big.2019.0169 32820952

[CR49] Scholtes I (2017) When is a network a network? Multi-order graphical model selection in pathways and temporal networks. In: Proceedings of the 23rd ACM SIGKDD international conference on knowledge discovery and data mining. ACM, pp 1037–1046

[CR50] Schwarze AC, Porter MA (2021). Motifs for processes on networks. SIAM J Appl Dyn Syst.

[CR51] Shapira SD, Gat-Viks I, Shum BO, Dricot A, Grace MM, Wu L, Gupta PB, Hao T, Silver SJ, Root DE (2009). A physical and regulatory map of host-influenza interactions reveals pathways in h1n1 infection. Cell.

[CR52] Singer P, Helic D, Taraghi B, Strohmaier M (2014). Detecting memory and structure in human navigation patterns using Markov chain models of varying order. PLoS ONE.

[CR53] Tax N, Teinemaa I, Zelst SJ (2020). An interdisciplinary comparison of sequence modeling methods for next-element prediction. Softw Syst Model.

[CR54] Torres L, Blevins AS, Bassett D, Eliassi-Rad T (2021). The why, how, and when of representations for complex systems. SIAM Rev.

[CR55] Transport for London: Rolling Origin and Destination Survey (RODS) database (2014). http://www.tfl.gov.uk/info-for/open-data-users/our-feeds

[CR56] Wang L, Wu JT (2018). Characterizing the dynamics underlying global spread of epidemics. Nat Commun.

[CR57] Weijters A, Ribeiro J (2011) Flexible heuristics miner (fhm). In: 2011 IEEE symposium on computational intelligence and data mining (CIDM). IEEE, , pp 310–317

[CR58] West R, Leskovec J (2012) man wayfinding in information networks. In: Proceedings of the 21st International conference on World Wide Web, pp 619–628

[CR59] Xu J, Wickramarathne TL, Chawla NV (2016) Representing higher-order dependencies in networks. Sci Adv 10.1126/sciadv.1600028 PMC4928957 27386539

